# Flat-on ambipolar triphenylamine/C_60_ nano-stacks formed from the self-organization of a pyramid-sphere-shaped amphiphile†
†Electronic supplementary information (ESI) available: ^1^H and ^13^C NMR spectra, TGA, DSC, XRD, simulated ED patterns, UV-Vis spectra, reduction and oxidation cyclic voltammetry curves. See DOI: 10.1039/c5sc04242a


**DOI:** 10.1039/c5sc04242a

**Published:** 2016-01-04

**Authors:** Wei-Wei Liang, Chi-Feng Huang, Kuan-Yi Wu, San-Lien Wu, Shu-Ting Chang, Yen-Ju Cheng, Chien-Lung Wang

**Affiliations:** a Department of Applied Chemistry , National Chiao Tung University , 1001 Ta Hsueh Road , Hsin-Chu , 30010 , Taiwan . Email: kclwang@nctu.edu.tw

## Abstract

A giant amphiphile, which is constructed with an amorphous nano-pyramid (triphenylamine, TPA) and a crystalline nano-sphere (C_60_), was synthesized. Structural characterization indicates that this pyramid-sphere-shaped amphiphile (**TPA–C_60_**) forms a solvent-induced ordered phase, in which the two constituent units self-assemble into alternating stacks of two-dimensional (2D) TPA and C_60_ nano-sheets. Due to the complexity of the molecular structure and the amorphous nature of the nano-pyramid, phase formation was driven by intermolecular C_60_–C_60_ interactions and the ordered phase could not be reformed from the **TPA–C_60_** melt. Oriented crystal arrays of **TPA–C_60_**, which contain flat-on TPA/C_60_ nano-stacks, can be obtained *via* a PDMS-assisted crystallization (PAC) technique. The flat-on dual-channel supramolecular structure of **TPA–C_60_** delivered ambipolar and balanced charge-transport characteristics with an average *μ*_e_ of 2.11 × 10^–4^ cm^2^ V^–1^ s^–1^ and *μ*_h_ of 3.37 × 10^–4^ cm^2^ V^–1^ s^–1^. The anisotropic charge-transport ability of the pyramid-sphere-shaped amphiphile was further understood based on the lattice structure and the lattice orientation of **TPA–C_60_** revealed from electron diffraction analyses.

## Abstract 2

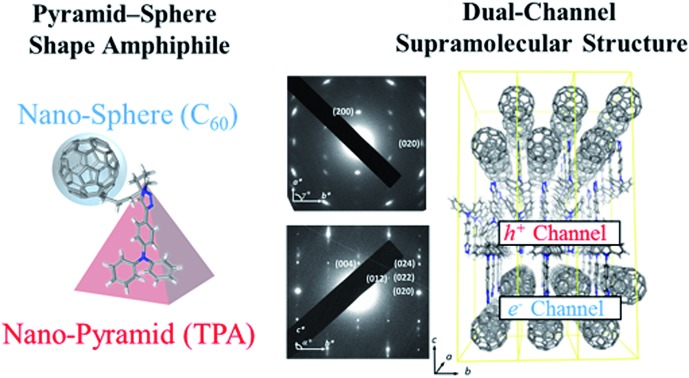
The study identified the flat-on dual-channel nano-structure and the ambipolar characteristics of a novel giant pyramid-sphere shape amphiphile.

## 1. Introduction

Giant amphiphiles are synthesized by covalently binding various nm-sized molecular building blocks (or so-called molecular nanoparticles (MNPs)).^1^ Their self-assembly processes are driven by competitive or cooperative physical interactions among the MNPs and are largely influenced by the preferred packing scheme of the constituent MNPs. The giant amphiphiles contain a wide range of emerging materials^1^,^2^ such as sphere-cube,^1^ sphere-board,^3^–^5^ sphere-disc,^6^–^19^ discotic-rod,^20^,^21^ cube-disk,^22^ and cube-board^23^-shaped amphiphiles. Among these giant molecules, those built with p-type and n-type conjugated moieties have attracted much attention, because of their potential applications as active units in supramolecular optoelectronics.^5^,^24^,^25^

**Scheme 1:**
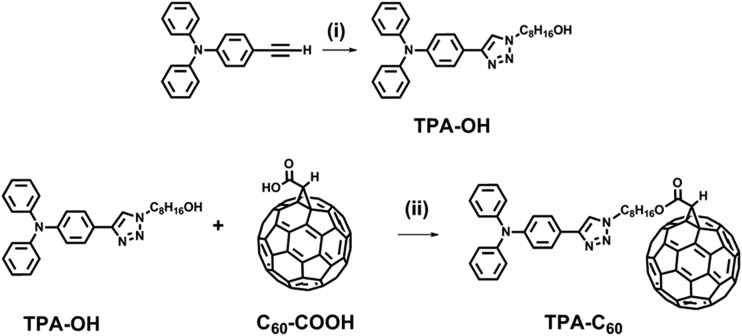
Synthetic route of **TPA–C_60_**. Reagents and conditions: (i) 8-azidooctan-1-ol, copper(ii) sulfate pentahydrate, sodium ascorbate, THF/H_2_O (1/1, v/v); (ii) *p*-toluenesulfonic acid, 4-dimethylaminopyridine, 1-(3-dimethylaminopropyl)-2-ethylcarbodiimide hydrochloride, CS_2_.

Giant molecules can be constructed from crystalline mesogenic, quasicrystalline and amorphous MNPs,^26^ but so far, most giant amphiphiles are made with MNPs that are intrinsically crystalline. Triphenylamine (TPA)-based conjugated molecules are widely used in organic light emitting diodes (OLEDs),^27^,^28^ polymer solar cells (PSCs),^29^ and perovskite solar cells,^30^ because of their good morphological stability and p-type semiconducting characteristics. However, they are rarely reported as MNPs of giant amphiphiles, probably due to their nonplanar structure and amorphous nature.^27^,^28^ The incorporation of this pyramidal nano-building block raises interesting questions, such as whether the self-organization of a giant amphiphile will be compromised due to the presence of an amorphous nano-pyramid, or on the other hand, whether the amorphous nano-pyramid will self-organize because the other MNP of the giant amphiphile tends to crystallize. To explore these questions, a giant pyramid-sphere-shaped amphiphile, **TPA–C_60_**, which is constructed with the amorphous nano-pyramid (TPA) and the crystalline nano-sphere (C_60_), was designed and synthesized based on Steglich esterification and copper-catalyzed azide–alkyne cycloaddition (CuAAC), as shown in Scheme 1. The phase behavior and phase structure of **TPA–C_60_** were investigated *via* differential scanning calorimetry (DSC) and electron diffraction (ED). The field effect transistor (FET) characteristics of **TPA–C_60_** were evaluated with oriented **TPA–C_60_** crystal arrays prepared *via* the PDMS-assisted crystallization (PAC) method.^31^ The results show that the pyramid-sphere-shaped amphiphile self-organized into an ordered phase containing stacks of alternating two-dimensional (2D) C_60_ and TPA nanosheets. Moreover, this dual-channel supramolecular structure of **TPA–C_60_** delivered ambipolar and balanced charge-transport characteristics in organic field-effect transistors (OFETs). Previous studies showed that TPA and C_60_ have preferable physical interactions and tend to form a mixed TPA/C_60_ domain in the solid-state.^32^–^34^ However, with respect to functions, the mixed TPA/C_60_ domain is not conducive to ambipolar charge transport. Our study thus gives the first example of flat-on ambipolar TPA/C_60_ nano-stacks obtained *via* the self-organization of a pyramid-sphere-shaped amphiphile.

## 2. Results and discussion

### 2.1. Synthesis of **TPA–C_60_**

Scheme 1 shows the synthetic route of **TPA–C_60_**. 4-Ethynyl-*N*,*N*-diphenylaniline, 8-azidooctan-1-ol, and fullerenoacetic acid (**C_60_-COOH**) were synthesized according to the literature.^35^ Reacting 4-ethynyl-*N*,*N*-diphenylaniline with 8-azidooctan-1-ol *via* the CuAAC reaction allowed the formation of a p-type pyramid unit, 8-(4-(triphenylamino)-1*H*-1,2,3-triazol-1-yl)octan-1-ol (**TPA-OH**) in 67% yield. The final product, **TPA–C_60_**, was then synthesized in 74% yield by reacting **TPA-OH** and **C_60_-COOH***via* Steglich esterification. **TPA–C_60_** was characterized by ^1^H NMR, ^13^C NMR, and mass spectrometry. As shown in Fig. S1,† the formation of **TPA–C_60_** was identified by the downfield shift of the methylene protons of **TPA-OH** (denoted as H_a_ in Fig. S1†) and the appearance of a methine proton (H_b_ in Fig. S1b†) at *δ* = 4.88 ppm, which belongs to the C_60_ moiety. The multiple peaks between *δ* = 135 and 145 ppm in the ^13^C-NMR spectrum of **TPA–C_60_** (Fig. S2b†), are also characteristic of the sp^2^ carbons on the mono-adduct C_60_ moiety. Furthermore, as shown in Fig. S3,† the [M + H]^+^ peak of the final product has an *m*/*z* value of 1201.267, which matches well with the calculated monoisotopic mass (1201.24 Da). All the results clearly indicate the success of the reaction and confirm the chemical identity and purity of **TPA–C_60_**.

### 2.2. Thermal stability and phase transition

After vacuum drying using a cryo pump, thermogravimetric analysis (Fig. S4a†) showed a 5% weight loss temperature of **TPA–C_60_** at 388 °C. As shown in Fig. S4b,† an additional weight loss peak at 131 °C was observed for a **TPA–C_60_** sample that was only vacuum dried under a mechanical pump. The results indicate that the as-precipitated **TPA–C_60_** contains residual solvent molecules that can be removed under high vacuum. DSC was then applied to identify the phase behaviour of the two MNPs and the pyramid-sphere-shaped amphiphile. As shown in Fig. S5a,† 4-ethynyl-*N*,*N*-diphenylaniline shows an endothermic first-order transition at 108 °C during the 1^st^ heating, suggesting that the as-precipitated sample self-organized into an ordered phase. However, a corresponding exothermic transition in the subsequent cooling was not observed, indicating that 4-ethynyl-*N*,*N*-diphenylaniline was vitrified rather than crystallized during the cooling process. Consequently, in the 2^nd^ heating (Fig. S5b†), the endothermic transition at 108 °C disappeared. As shown in Fig. S5c,† **TPA-OH** exhibits only a glass transition temperature at 10 °C. Thus, the results show the easily disturbed self-organization behaviour of the TPA unit, *i.e.* although 4-ethynyl-*N*,*N*-diphenylaniline self-organizes from the solution, this behaviour is lost in the melt and when a flexible alkyl group is attached. For **TPA–C_60_**, three first-order transitions at 190, 226 and 239 °C were found in the 1^st^ heating curve of **TPA–C_60_** (Fig. S5d†), indicating that **TPA–C_60_** packs into an ordered solid-state structure. The multiple transitions suggest that instead of directly transforming into the isotropic melt, the ordered phase of **TPA–C_60_** may lose its structural order (conformational, orientational, and positional orders) in a stepwise way. In the cooling curve, no exothermic peak was observed, indicating that although self-organization of **TPA–C_60_** from solution is possible, reforming the ordered packing from the melt is difficult, probably due to the structural complexity of **TPA–C_60_**. In short, the DSC results revealed the amorphous nature of the alkylated TPA nano-pyramid and the crystalline characteristics of the **TPA–C_60_** amphiphile. More importantly, it was found that the amorphous alkylated nano-pyramid (TPA) can self-assemble under the assistance of favourable intermolecular interactions among the crystalline nano-spheres (C_60_).

### 2.3. Phase morphology of **TPA–C_60_**

**Fig. 1:**
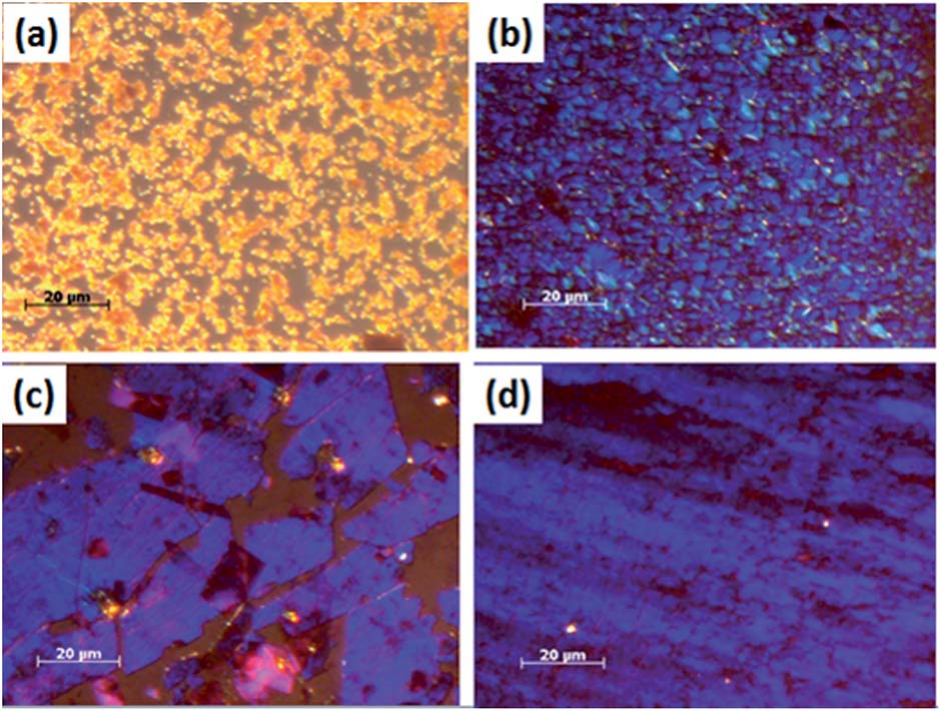
POM images of **TPA–C_60_** processed *via* the PAC method with different solvents. (a) *m*-Xylene, (b) CS_2_, (c) ODCB, and (d) TCB.

**TPA–C_60_** tends to form tiny crystals in drop-cast thin films. To obtain crystals large enough for device fabrication and to introduce better crystal orientation, the PAC method^31^ was applied to produce a crystal array of **TPA–C_60_**. Fig. 1 shows **TPA–C_60_** films obtained from different solvents. As shown in Fig. 1a and b, the crystal sizes were small when **TPA–C_60_** was processed from *m*-xylene and CS_2_ solutions. However, the bright blue or yellow POM images suggested that **TPA–C_60_** has birefringence and structural order in the cast films. Large crystalline **TPA–C_60_** sheets were obtained from *o*-dichlorobenzene (ODCB) and 1,2,4-trichlorobenzene (TCB) solutions (Fig. 1c and d). Intriguingly, as shown in Fig. S6,† the color of the crystalline sheet was only observed when the incident light was polarized (Fig. S6c and d†) and the crystal does not turn dark even when its growth direction is along the polarization direction of the polarizer or the analyzer. A similar phenomenon was observed in the blue phases of highly chiral liquid crystals.^36^ The origin of the blue or yellow POM images of **TPA–C_60_** falls outside the scope of the current study and will be investigated separately.

**Fig. 2:**
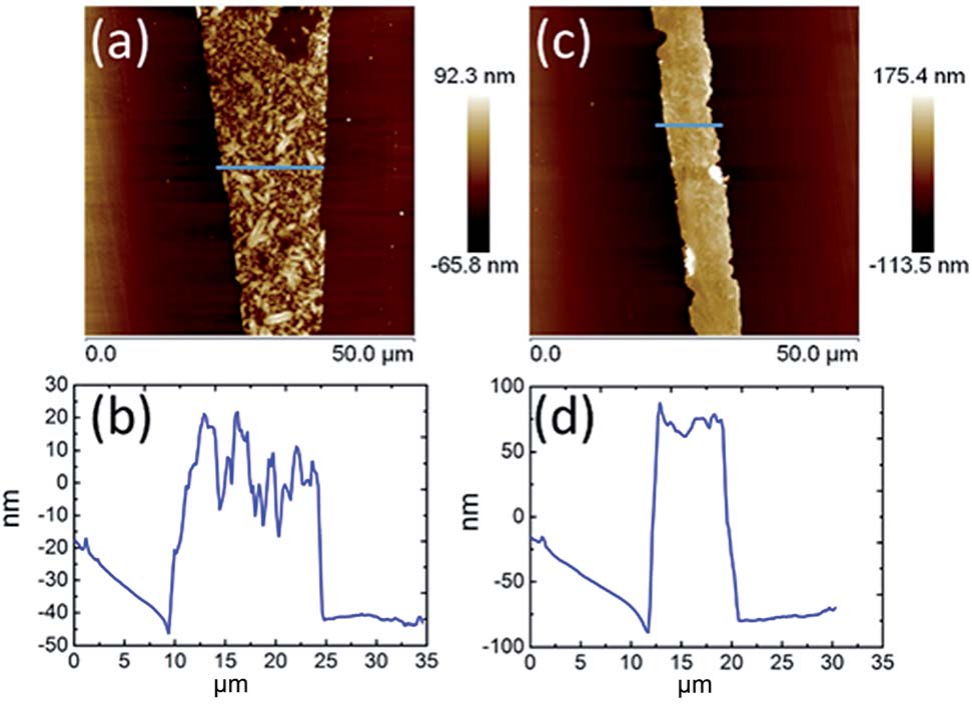
AFM topography and cross-section profiles of **TPA–C_60_** processed *via* the PAC method with ODCB (a) and (b) and TCB (c) and (d).

**Fig. 3:**
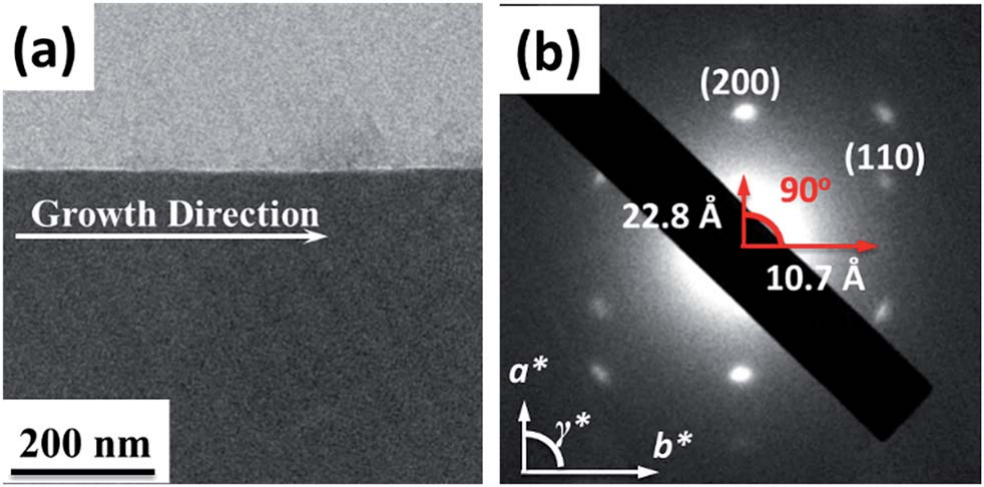
(a) TEM image and (b) the enlarged ED pattern of the **TPA–C_60_** crystal array prepared *via* the PAC method using TCB as solvent.

The AFM topography and cross-section profiles in Fig. 2 show that the **TPA–C_60_** film prepared from ODCB solution is a polycrystalline thin film with a very rough surface (max. thickness ∼ 80 nm), but the one prepared from TCB solution has an insignificant crystalline boundary and a uniform surface (thickness ∼ 150 nm). The crystal array prepared from TCB was further examined with transmission electron microscopy (TEM) and electron diffraction (ED). Similar to the AFM result, the **TPA–C_60_** crystal has a uniform appearance in the TEM image (Fig. 3a). The clear diffraction spots in the ED pattern (Fig. 3b) are evidence of the formation of an ordered solid-state structure, and more importantly, the well-oriented crystal lattices in the **TPA–C_60_** crystal array formed by the PAC method. These morphological characterization methods thus confirmed that the pyramid-sphere-shaped amphiphile, **TPA–C_60_**, can assemble into an ordered phase, and a good lattice orientation can be induced *via* the PAC method.

### 2.4. Phase structure of **TPA–C_60_**

**Fig. 4:**
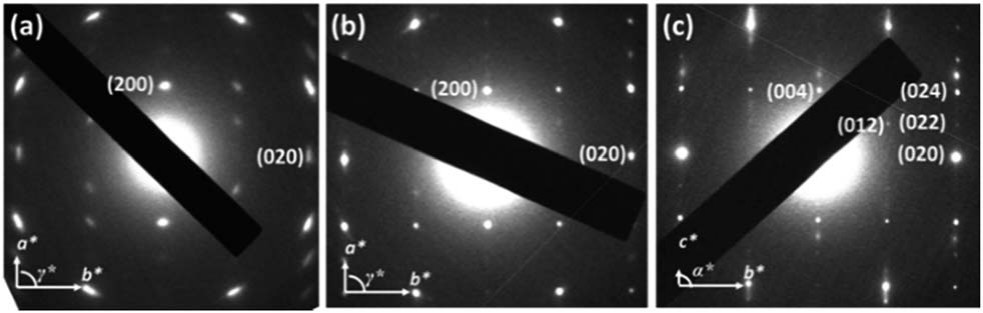
ED patterns of **TPA–C_60_** crystal prepared from (a) the PAC method, (b) and (c) by drop-casting. Projections of the a*b* and the b*c* reciprocal lattices can be observed in (b) and (c).

**Fig. 5:**
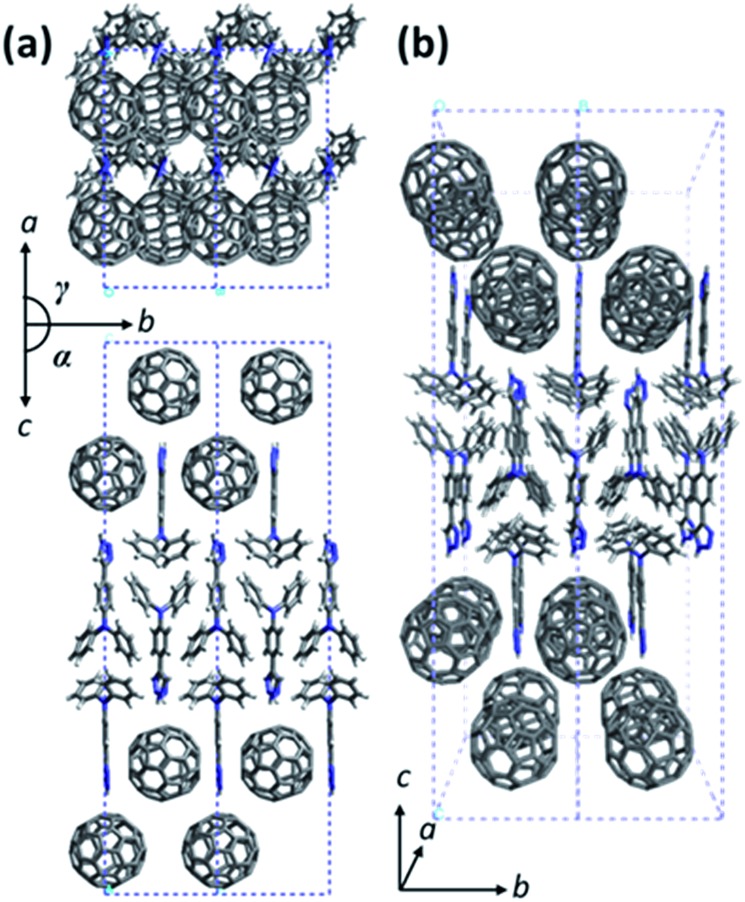
The lattice model of **TPA–C_60_**. (a) The *ab* and the *bc* lattice projections and (b) the projection view of the lattice.

Due to the structural complexity, preparation of a large single crystal of **TPA–C_60_** for single-crystal structural characterization was difficult. In addition, the ED patterns along different zone axes are still useful for revealing the 2D lattice projections of the **TPA–C_60_** crystal. Using the drop-cast sample, a*b* and the b*c* reciprocal lattices (Fig. 4b and c) were observed. The lattice parameters deduced from Fig. 4b are *a* = 22.8 Å, *b* = 10.7 Å, *γ* = 90°; and from Fig. 4c are *b* = 10.7 Å, *c* = 51.0 Å, and *α* = 90°. The measured density of the **TPA–C_60_** crystal is 1.28 g cm^–3^. Assuming that *β* = 90°, the density provides the information that the orthorhombic lattice (*a* = 22.8 Å, *b* = 10.7 Å, *c* = 51.0 Å, *α* = *β* = *γ* = 90°) contains 8 **TPA–C_60_** molecules per unit cell. The powder X-ray diffraction (XRD) pattern of **TPA–C_60_** is shown in Fig. S7.† The theoretical *d*-spacings of various lattice planes calculated from the abovementioned lattice parameters also match the measured ones (Table S1†). Furthermore, the (100) and (010) diffractions in Fig. 4b are significantly weaker than the (200) and (020) diffractions, suggesting that in the lattice, the electron density on the (100) planes is close to that on the (200) planes and that on the (010) planes is similar to that on the (020) planes. A lattice model was then built using the Cerius^2^ software package, based on the abovementioned information, and is shown in Fig. 5. The simulated ED patterns (Fig. S8†) generated from the [001] and [100] zones of the model resemble the experimental ones in Fig. 5, showing the validity of the model. The lattice of **TPA–C_60_** features a dual-channel structure containing continuous but separated 2D n-type C_60_ sheets and p-type TPA sheets (Fig. 5b). It is noteworthy that the C_60_-to-C_60_ distance along the *a* axis (11.4 Å) is different from that along the *b* axis (10.7 Å). The ED pattern of the crystal array prepared by PAC (Fig. 4a) shares the zone axis, [001], with the pattern in Fig. 4b. Thus, it is confirmed that in the PAC method, the growth direction of the crystal array is along the *b* axis of the **TPA–C_60_** lattice, and the 2D C_60_ and TPA sheets adopt a flat-on orientation of the substrate.

### 2.5. Optical and electrochemical properties

The UV-Vis absorption spectrum and cyclic voltammogram (CV) of **TPA–C_60_** are shown in Fig. S9 and S10.† For comparison, the absorption spectra and the CVs of [6,6]-phenyl-C_61_-butyric acid methyl ester (PCBM) and **TPA-OH** are also included in the figures. As shown in Fig. S6,† the absorption maximum of **TPA–C_60_** is similar to that of PCBM. The slightly stronger absorption at 315 nm was attributed to the TPA moiety of **TPA–C_60_**. Fig. S10† shows three reversible reductions and one oxidation. The reduction potential, oxidation potential, and HOMO and LUMO energies of the three compounds are summarized in Table S2.† The HOMO and LUMO energies of **TPA–C_60_** are close to the HOMO energy of **TPA-OH** and the LUMO energy of PCBM, suggesting that the C_60_ and TPA moieties of **TPA–C_60_** retain their individual characteristics in the molecule.

### 2.6. OFET performance

**Fig. 6:**
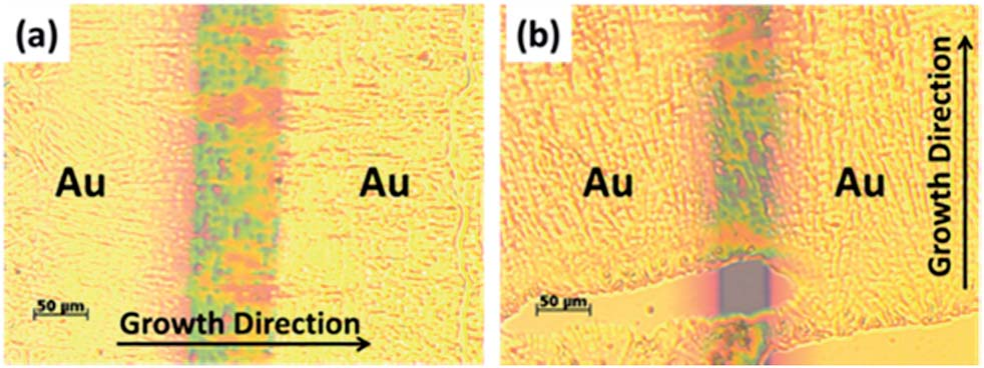
OM images of the **TPA–C_60_** OFET devices with the gold (Au) source and drain electrodes arranged (a) parallel and (b) perpendicular to the crystal growth direction.

**Fig. 7:**
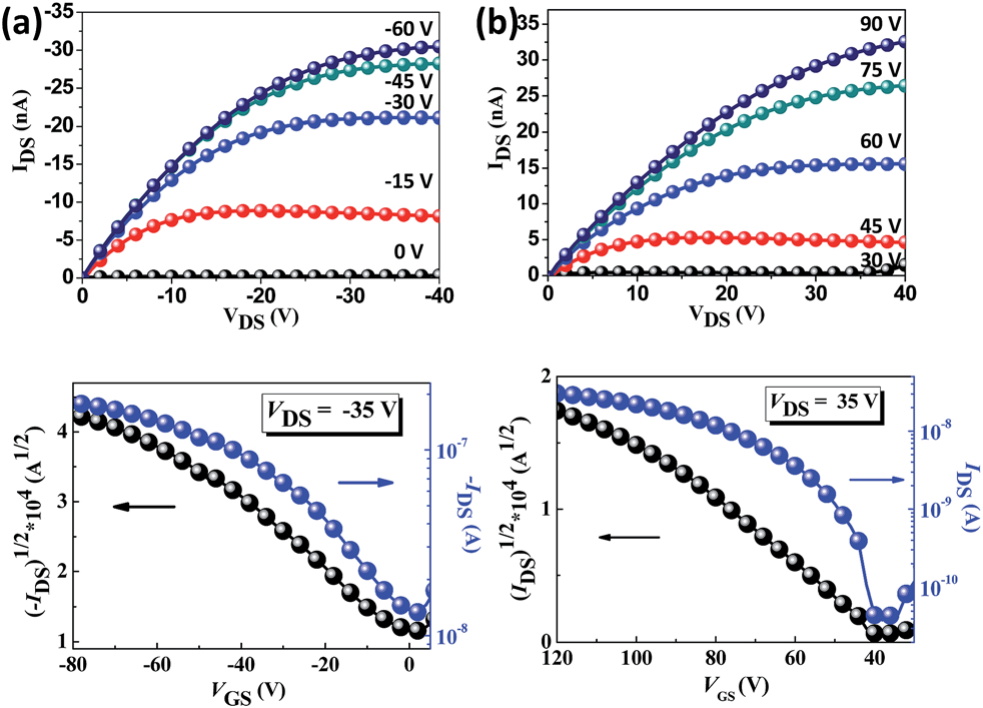
The output (up) and transfer (down) characteristics of the **TPA–C_60_** crystal arrays under (a) negative *V*_GS_ and (b) positive *V*_GS_.

The charge transport properties of the oriented **TPA–C_60_** crystal arrays prepared by the PAC method were investigated in OFET devices with a bottom-gate, top-contact configuration. Because the ED results confirmed that the *b* lattice axis of **TPA–C_60_** is parallel to the crystal growth direction induced by PAC, as shown in Fig. 6, the source and drain electrodes were arranged parallel or perpendicular to the growth direction, so that the anisotropic charge-transport characteristics of the **TPA–C_60_** crystal arrays along the *a* and *b* axes could be studied. The output and transfer plots of the best-performing device are shown in Fig. 7. The **TPA–C_60_** crystal array demonstrated the p-channel characteristics under a negative gate-to-source voltage (*V*_GS_) (Fig. 7a) and the n-channel characteristics under a positive *V*_GS_ (Fig. 7b). The hole mobilities (*μ*_h_s) and electron mobilities (*μ*_e_s) deduced from the transfer plots of the devices in saturation regimes are summarized in Table 1. The averaged *μ*_h_s values are 3.37 × 10^–4^ cm^2^ V^–1^ s^–1^ for the parallel devices and 5.43 × 10^–5^ cm^2^ V^–1^ s^–1^ for the perpendicular devices. In the database we have searched, the highest OFET *μ*_h_ of a TPA-star burst conjugated molecule was around 3 × 10^–4^ cm^2^ V^–1^ s^–1^.^37^ Thus, the 2D sheets of TPA in the **TPA–C_60_** crystal array retained their hole-transporting ability and delivered one of the best *μ*_h_ values among the triarylamine-based molecules. On the other hand, the 2D sheets of the mono-adduct C_60_ delivered averaged *μ*_e_s values of 2.11 × 10^–4^ cm^2^ V^–1^ s^–1^ for the parallel devices and 3.54 × 10^–5^ cm^2^ V^–1^ s^–1^ for the perpendicular devices. Thus, the **TPA–C_60_** crystal array has ambipolar charge transport characteristics and delivers balanced hole and electron charge mobilities. Moreover, the anisotropic charge-transport characteristics of the **TPA–C_60_** crystal array are obvious, because the *μ*_h_ and *μ*_e_ values along the growth direction (*b* lattice axis) are about one order of magnitude higher than those perpendicular to the growth axis (*a* lattice axis). The difference can be attributed to shorter TPA–TPA and C_60_–C_60_ distances along the *b* axis, as shown in the *ab* lattice projection in Fig. 5a. The lower *μ*_e_ of the **TPA–C_60_** crystal array compared to the best-performing C_60_ OFETs may be related to the longer C_60_–C_60_ distance and the lower coordination number of C_60_s in the **TPA–C_60_** crystal lattice.^31^,^38^ In addition, the smeared ED pattern in Fig. 4a suggests a certain degree of orientational disorder in the crystal array, which is also detrimental to the *μ*_e_.

**Table 1:** OFET characteristics of the **TPA–C_60_** crystal arrays^*a*^

	*μ*_e average_ (cm^2^ V^–1^ s^–1^)	*I*_on_/*I*_off_	*V*_th_ (V)	*μ*_h average_ (cm^2^ V^–1^ s^–1^)	*I*_on_/*I*_off_	*V*_th_ (V)
Parallel	2.11 × 10^–4^	3 × 10^4^	32	3.37 × 10^–4^	6 × 10^3^	–22
Perpendicular	3.54 × 10^–5^	1 × 10^1^	16	5.43 × 10^–5^	2 × 10^3^	–15

^*a*^The *μ*s values of the parallel devices were averaged from 8 devices, and those of the perpendicular device were averaged from 6 devices.

## 3. Conclusions

In summary, a synthetic route for a giant amphiphile (**TPA–C_60_**), which is constructed with an amorphous pyramid (TPA) and a crystalline sphere (C_60_), was developed. DSC results show that the pyramid-sphere-shaped amphiphile forms a solvent-induced ordered phase, because of the strong crystalline nature of the C_60_ moiety. Structural characterization confirmed that **TPA–C_60_**s self-assemble into separated 2D C_60_ and TPA sheets in the ordered phase. Processing by the PAC method produced oriented crystal arrays of **TPA–C_60_**. ED results indicate that the 2D C_60_ and TPA sheets adopt a flat-on orientation on the substrate with the *b* lattice axis pointing in the crystal growth direction. The flat-on n-type C_60_ nano-sheets and p-type TPA nano-sheets provide electron and hole-transporting channels. Ambipolar and balanced charge-transport characteristics were delivered by the **TPA–C_60_** crystal arrays in OFET devices. The dual-channel supramolecular structure of **TPA–C_60_** delivered an averaged *μ*_e_ of 2.11 × 10^–4^ cm^2^ V^–1^ s^–1^ and a *μ*_h_ of 3.37 × 10^–4^ cm^2^ V^–1^ s^–1^. The *μ*_h_ is comparable to the best-performing triarylamine-based p-type conjugated molecules, whereas the modest *μ*_e_ delivered by the C_60_ nano-sheets was attributed to the longer C_60_–C_60_ distance, the lower C_60_ coordination number in the **TPA–C_60_** crystal lattice and the less orientational order of the **TPA–C_60_** crystal array, compared to the pristine C_60_ crystals. Although a 2D crystal array of C_60_ has been recently disclosed,^39^ our study revealed the first example of a dual-channel self-organized structure and the ambipolar characteristics of a novel giant pyramid-sphere-shaped amphiphile.

## 4. Experimental section

### 4.1. General measurement and characterization

UV-Vis experiments were carried out using a HITACHI U-4100 spectrophotometer with a 10^–3^ M solution concentration in *o*-dichlorobenzene. The cyclic voltammetry (CV) data were analyzed with a CH Instruments Model 611D with a carbon glass serving as the working electrode and an Ag/Ag^+^ electrode as the reference electrode, with 0.1 M tetrabutylammonium hexafluorophosphate (Bu_4_NPF_6_) as the electrolyte and 10^–2^ M of the desired compound dissolved in *o*-dichlorobenzene. Thermogravimetric analysis (TGA) was conducted on a Perkin-Elmer Pyris under an inert atmosphere with a heating rate of 10 °C min^–1^ and differential scanning calorimetry (DSC) was performed on a TA Q200 Instrument at a temperature ramp rate of 5 °C min^–1^. For 1D XRD patterns, a Bruker APEX DUO single crystal diffractometer and an APEX II CCD camera equipped with a INCOATEC 18 kW rotating I microfocus X-ray generator (Cu Kα radiation (0.1542 nm)) were used. Transmission Electron Microscopy (TEM) observations were performed on a JEOL JEM-2010 transmission electron microscope with an accelerating voltage of 160 kV and a Gatan-831 CCD camera. Crystal simulation and drawing were based on a Cerius^2^ software product from Accelrys.

### 4.2. OFET fabrication

A n-type heavily doped Si wafer with a SiO_2_ layer of 300 nm and a capacitance of 11.5 nF cm^–2^ as the gate electrode and dielectric layer was ultrasonically cleaned sequentially in detergent, water and isopropyl alcohol. *N*-Octadecyltrichlorosilane (ODTS) was used as a self-assembled monolayer. The **TPA–C_60_** crystal arrays were prepared *via* the PDMS-assisted crystallization (PAC) method.^31^ The gold source and drain electrodes (40 nm in thickness) were then deposited on the organic layer by vacuum evaporation through a shadow mask, affording a bottom-gate, top-contact device configuration. OFET measurement was carried out at room temperature under a nitrogen atmosphere using an Agilent Technologies 4156C instrument. The mobility calculation was based on the equation *I*_ds_ = (*W*/2*L*)*μC*_i_(*V*_g_ – *V*_t_)^2^ in the saturation regime, where *I*_ds_ is the drain–source current, *W* is the channel width (1 mm), *L* is the channel length (100 *μ*m), *μ* is the field-effect mobility, *C*_i_ is the capacitance per unit area of the dielectric layer, *V*_g_ is the gate voltage, and *V*_t_ is the threshold voltage.

### 4.3. Synthesis

All chemicals were purchased from Aldrich, Acros or TCI and used as received unless specified otherwise. ^1^H and ^13^C NMR spectra were obtained in deuterium-substituted chloroform, CDCl_3_, as the reference with 0.5 wt% TMS, using Varian 400 MHz spectrometers.

### 4.4. 8-(4-(Triphenylamino)-1*H*-1,2,3-triazol-1-yl)octan-1-ol (**TPA-OH**)

To a solution of 4-ethynyl-*N*,*N*-diphenylaniline **3** (0.5 g, 1.86 mmol), copper(ii) sulfate pentahydrate (0.046 g, 0.184 mmol) and sodium ascorbate (0.11 g, 0.56 mmol) was added 8-azidooctan-1-ol **5** (0.382 g, 2.23 mmol) in THF/H_2_O 50 ml (1/1, v/v). The reaction mixture was stirred at room temperature for 3 hours then extracted with dichloromethane and water. The organic layer was collected and dried with MgSO_4_. After removal of the solvent under reduced pressure, the residue was purified by silica gel chromatography with ethyl acetate/hexane (1/3, v/v) as the eluent to give a beige solid (0.55 g, 67%). ^1^H NMR (400 MHz, CDCl_3_): *δ* 1.31–1.34 (m, 8H), 1.54 (t, 2H, *J* = 7 Hz), 1.93 (t, 2H, *J* = 7 Hz), 3.62 (t, 2H, *J* = 6.6 Hz), 4.37 (t, 2H, *J* = 7.2 Hz), 7.02 (t, 2H, *J* = 7.2 Hz), 7.12 (d, 6H), 7.24 (d, 2H), 7.26 (d, 2H), 7.66 (d, 2H), 7.69 (s, 1H). ^13^C NMR (CDCl_3_, 100 MHz): *δ* 25.6, 26.4, 28.9, 29.1, 30.3, 32.6, 50.3, 62.9, 118.8, 123.0, 123.8, 124.5, 124.7, 126.6, 129.3, 147.5, 147.7. MS (EI, C_28_H_32_N_4_O): calcd, 440.58; found, 440.5.

### 4.5. 8-(4-(Triphenylamino)-1*H*-1,2,3-triazol-1-yl)octyl acetate C_60_ (**TPA–C_60_**)

To a solution of **C_60_-COOH** (70 mg, 0.09 mmol), *p*-toluenesulfonic acid (17 mg, 0.09 mmol), 4-dimethylaminopyridine (11 mg, 0.09 mmol) and 1-(3-dimethylaminopropyl)-2-ethylcarbodiimide hydrochloride (17 mg 0.09 mmol) was added 8-(4-(triphenylamino)-1*H*-1,2,3-triazol-1-yl)octan-1-ol (60 mg, 13.6 mmol) in carbon disulfide (20 ml), the reaction mixture was stirred at room temperature for 12 hours. After removal of the solvent under reduced pressure, the residue was purified by neutral aluminum oxide chromatography with toluene to give a brown solid (79 mg, 74%). ^1^H NMR (400 MHz, CDCl_3_): *δ* 1.30–1.59 (m, 8H), 1.85 (t, 2H, *J* = 7.4 Hz), 1.95 (t, 2H, *J* = 7.4 Hz), 4.38 (t, 2H, *J* = 7.2 Hz), 4.45 (t, 2H, *J* = 6.6 Hz), 4.78 (s, 1H), 7.02 (t, 2H, *J* = 7.2 Hz), 7.10 (d, 6H), 7.24 (d, 2H), 7.26 (d, 2H), 7.67 (d, 2H), 7.69 (s, 1H). ^13^C NMR (CDCl_3_, 100 MHz): *δ* 25.9, 26.4, 28.6, 30.0, 30.4, 39.1, 50.3, 66.4, 70.6, 77.2, 118.8, 123.0, 123.8, 124.5, 124.8, 126.6, 129.3, 136.4, 140.4, 140.9, 141.1, 142.0, 142.1, 142.2, 142.4, 142.8, 143.0, 143.1, 143.2, 143.7, 143.9, 144.4, 144.6, 144.7, 145.0, 145.1, 145.2, 145.3, 145.6, 145.8, 147.5, 147.6, 147.7, 148.3, 166.4; MS (C_90_H_32_N_4_O_2_): calcd, 1201.24; found (FAB), 1200.9; found (MALDI-TOF), 1201.267.

## Supplementary Material

Supplementary information
