# Supplementary material for: Flat-on ambipolar triphenylamine/C60 nano-stacks formed from the self-organization of a pyramid-sphere-shaped amphiphile
Source: Chem Sci. 2016 Jan 4;7(4):2768–74. doi: 10.1039/c5sc04242a (PMC5477027; doi:10.1039/c5sc04242a)
Supplement: Supplementary file 1 [file SC-007-C5SC04242A-s001.pdf]

Electronic Supplementary Material (ESI) for Chemical Science.
This journal is © The Royal Society of Chemistry 2016

Supplementary information

The Flat-on Ambipolar Triphenylamine/C_{60} Nano-Stacks Formed from the Self-Organization of a Pyramid-Sphere Shape Amphiphile

Wei-Wei Liang,[a] Chi-Feng Huang,[a] Kuan-Yi Wu,[a] San-Lien Wu,[a] Shu-Ting Chang,[a] Yen-Ju Cheng,[a] and Chien-Lung Wang[a]*

[a]*Department of Applied Chemistry, National Chiao Tung University, 1001 Ta Hsueh Road Hsin-Chu, 30010, Taiwan*
E-Mail: kclwang@nctu.edu.tw

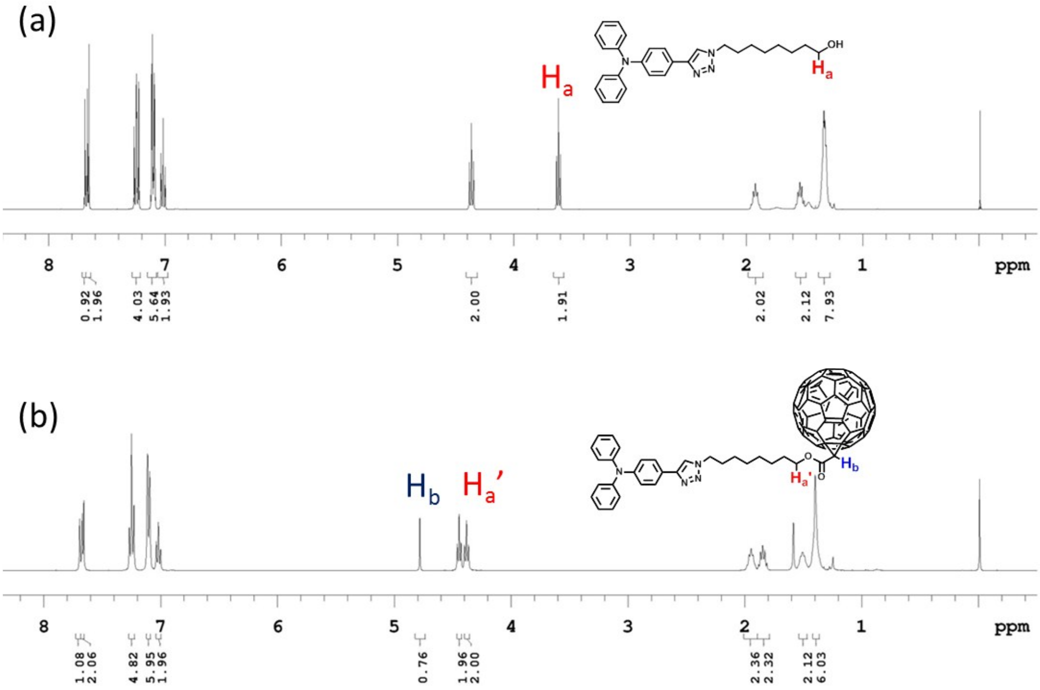

Fig. S1 ^{1}H NMR spectra of (a) TPA-OH and (b) **TPA-C_{60}**.

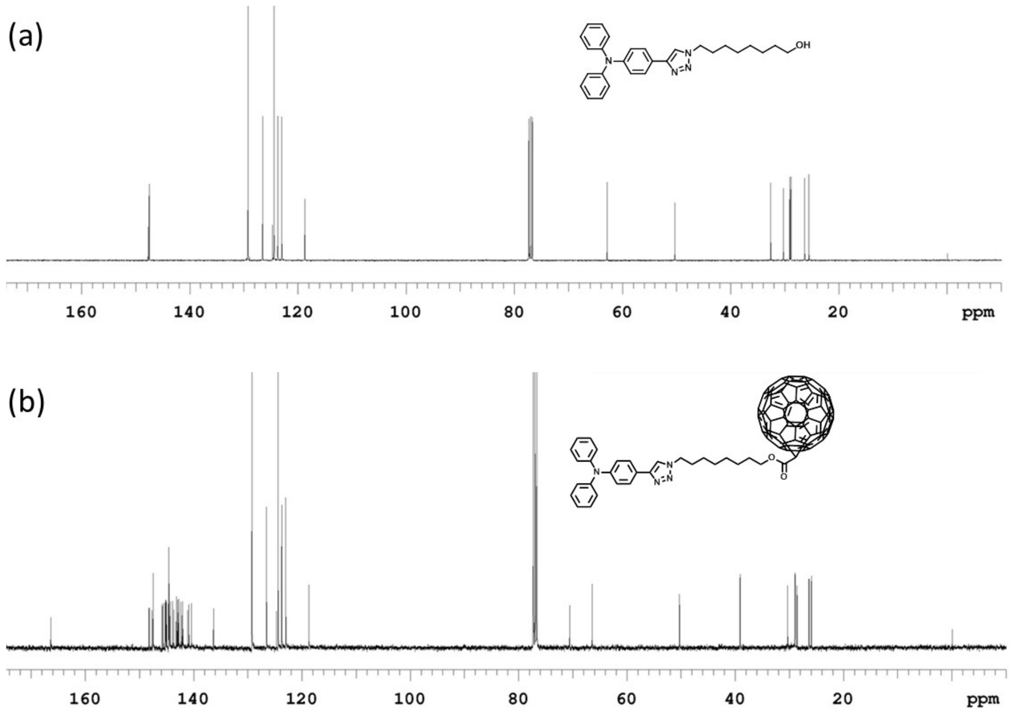

Fig. S2 ^{13}C NMR spectra of (a) TPA-OH and (b) **TPA-C_{60}**.

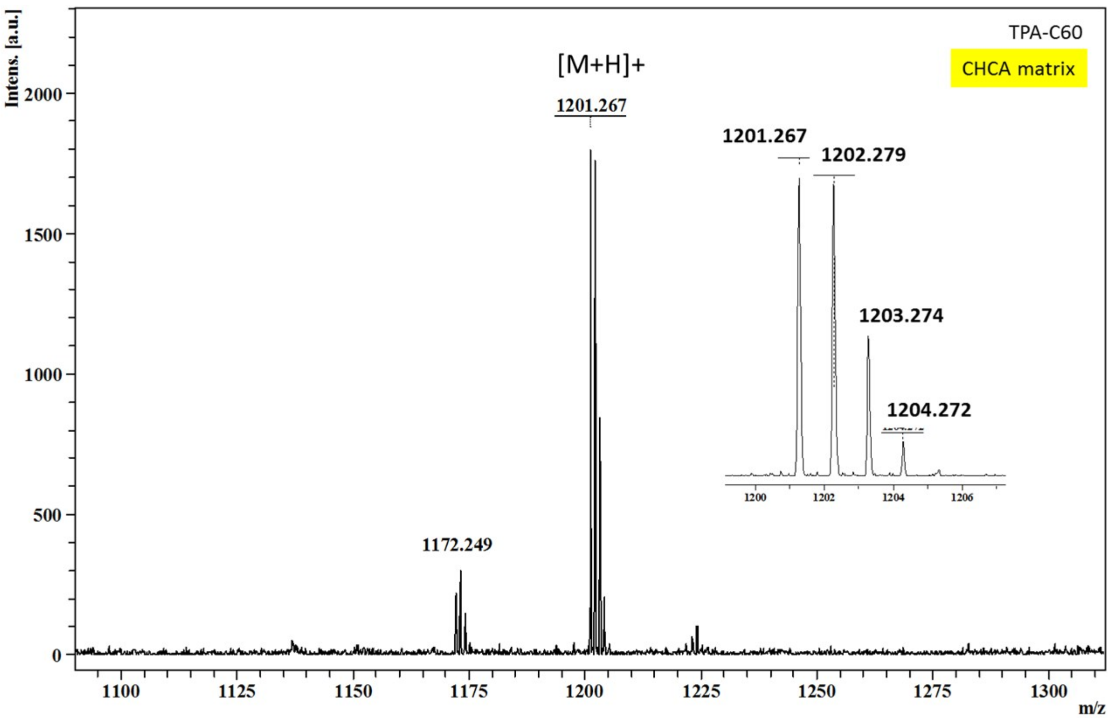

Fig. S3 MALDI TOF mass spectra of **TPA-C_{60}**.

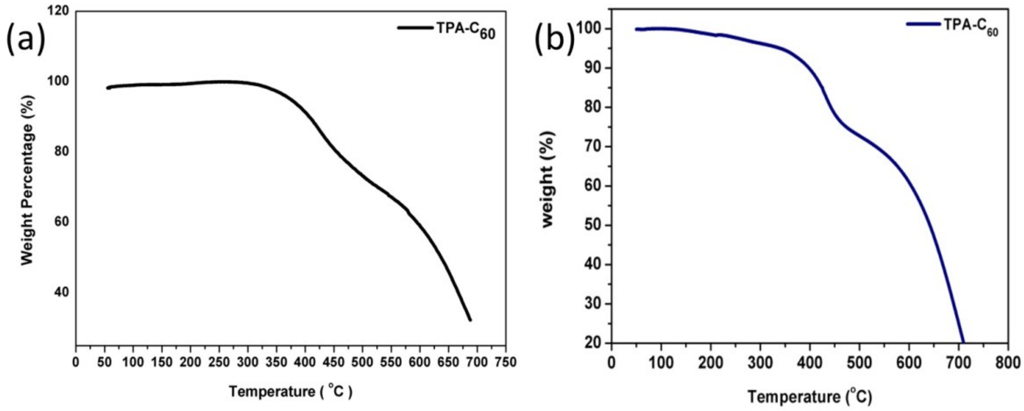

Fig. S4 Thermogravimetry analysis of the **TPA-C_{60}** samples dried by (a) a cryo pump (~ 6 × 10^{-7} torr) and (b) a mechanical pump (~10^{-2} torr).

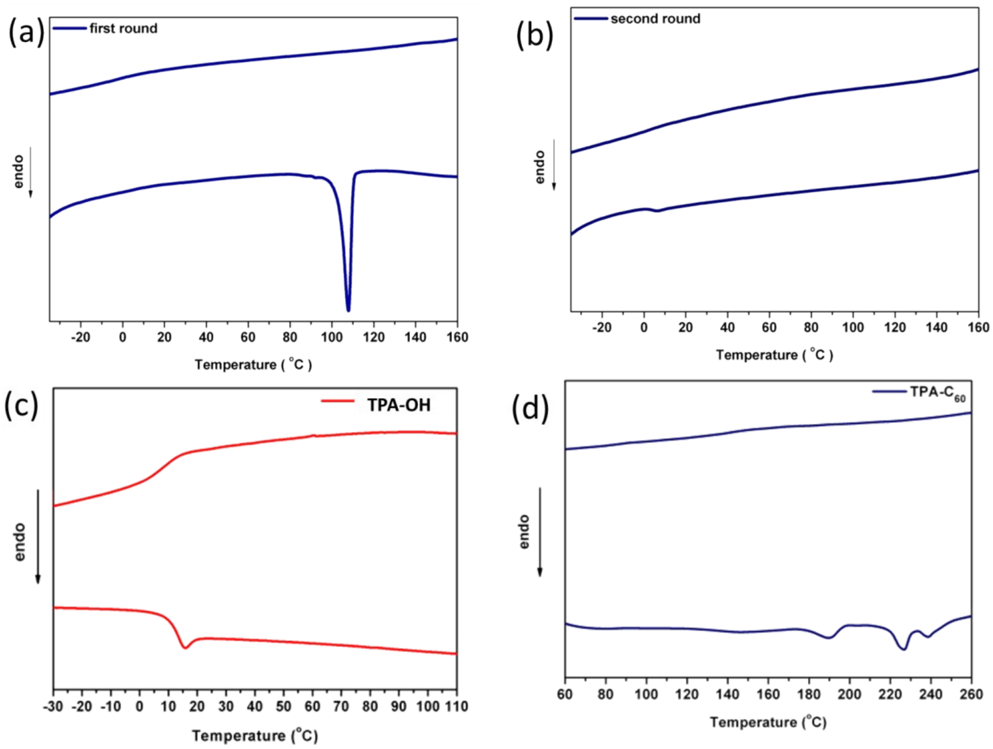

Fig. S5 DSC curves of (a) (b) 4-ethynyl-N,N-diphenylaniline, (c)**TPA-OH**, (d) **TPA-C_{60}**

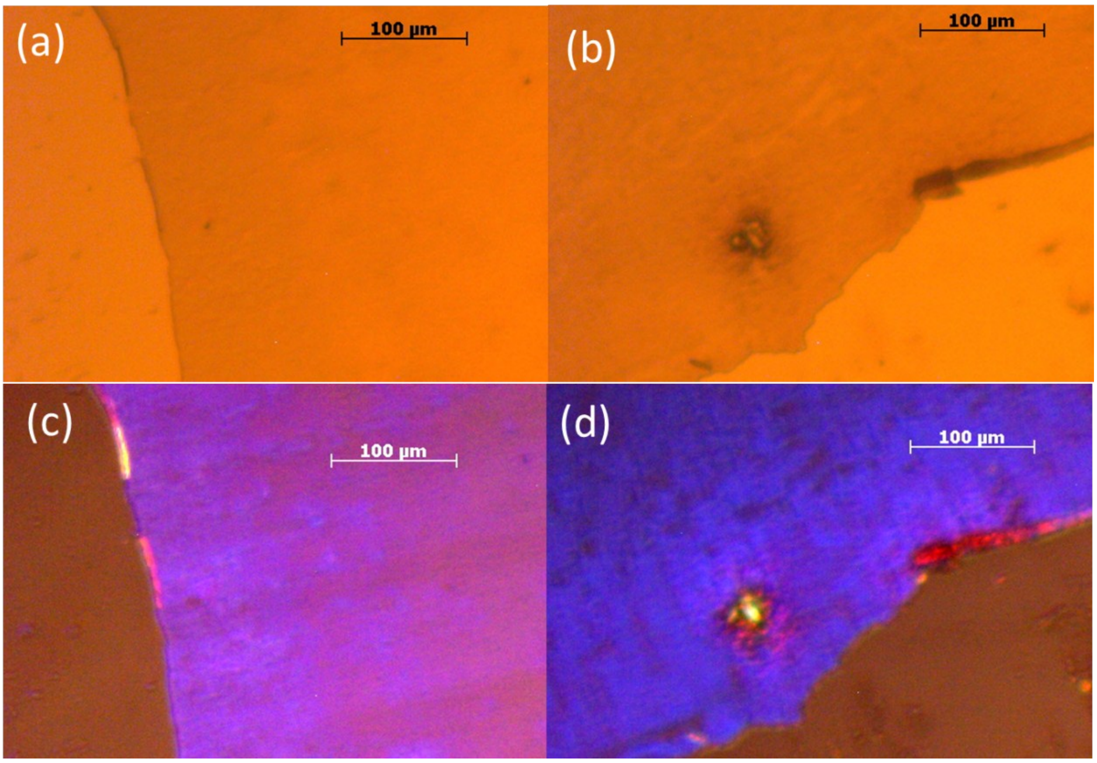

Fig. S6 Optical microscope (OM) images (a) (b), and POM images (c) (d) of **TPA-C_{60}** processed via the PAC method with ODCB.

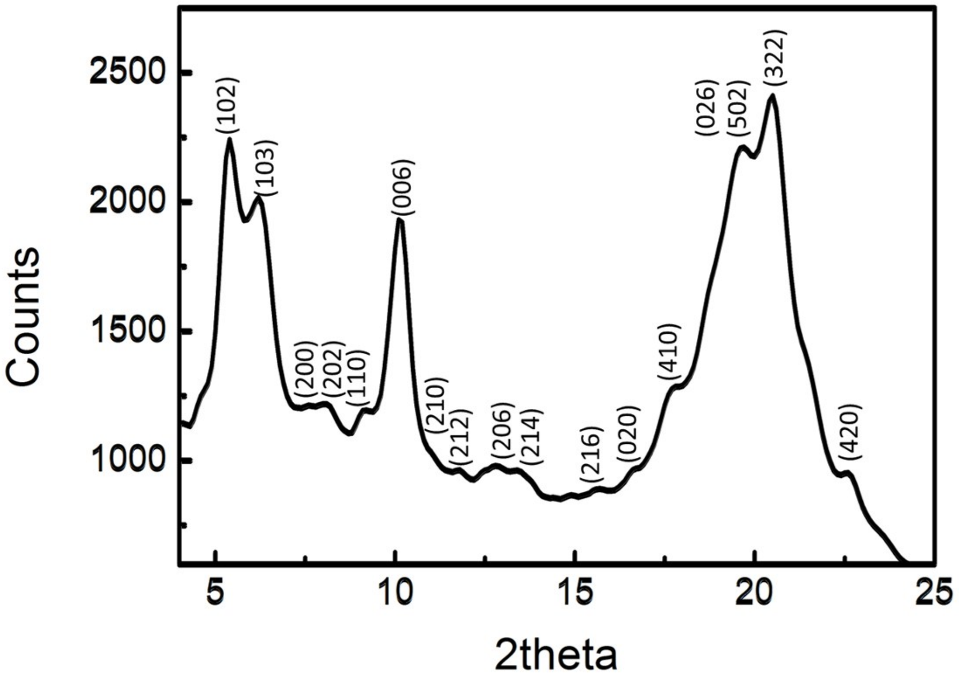

Fig. S7 The powder X-ray diffraction pattern of **TPA-C_{60}**.

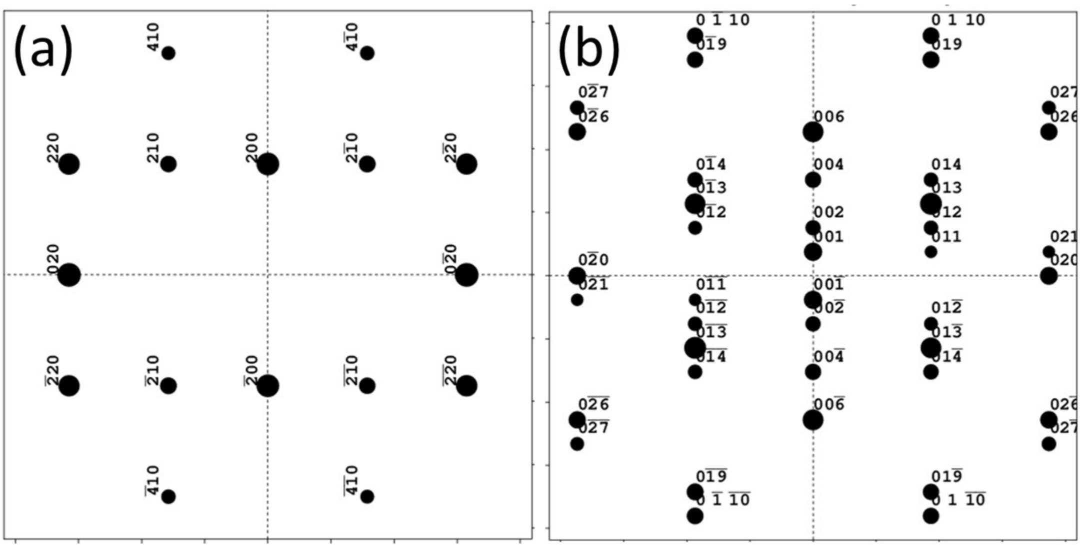

Fig. S8 Simulated ED patterns of **TPA-C_{60}** along (a) the [001] zone and (b) the [100] zone.

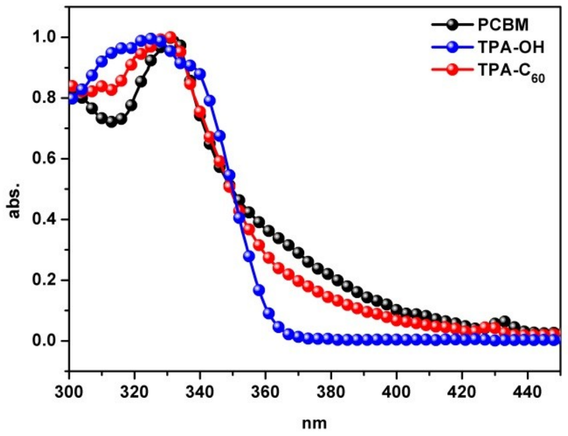

Fig. S9 UV-Vis spectra of **TPA-C_{60}**, TPA-OH, and PCBM.

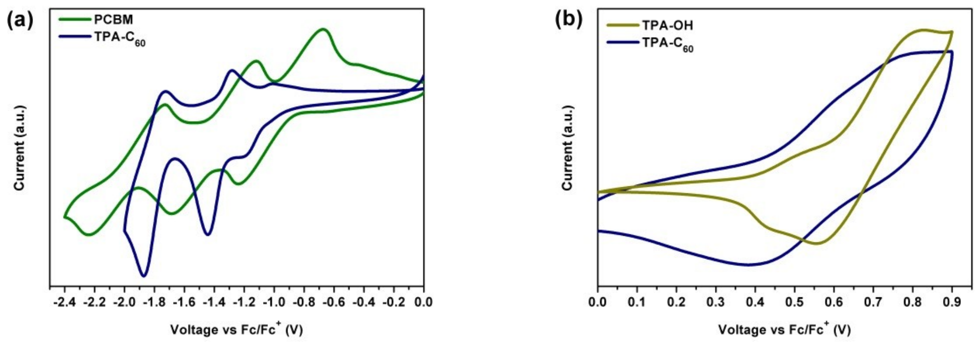

Fig. S10 (a) Reduction and (b) oxidation cyclic voltammetry curves of **TPA-C_{60}** and comparisons.

Table S1. The d-spacings of the (hkl) planes of the crystalline phase of **TPA-C_{60}**.

(hkl) plane	Theoretical d-spacing (Å)	Experimental d-spacing (Å)
(102)	16.4	16.3
(103)	14.2	14.2
(200)	11.4	11.6
(202)	10.4	10.9
(110)	9.7	9.7
(006)	8.5	8.7
(210)	7.8	8.0
(210)	7.5	7.5
(206)	6.8	6.9
(214)	6.7	6.6
(216)	5.7	5.7
(020)	5.4	5.3
(410)	5.0	5.0
(026)	4.5	4.5
(502)	4.5	4.5
(322)	4.3	4.3
(420)	3.9	3.9

Table S2. Reduction potential, oxidized potential, HOMO and LUMO energy of $PC_{61}BM$, **TPA-C_{60}** and TPA-OH.

	E_{ox}^{onset} [V]	E_{re}^{onset} [V]	HOMO [eV]	LUMO [eV]
**$PC_{61}BM$**		−0.88		−3.91
**TPA-C_{60}**	0.41	−0.85	−5.20	−3.94
**TPA-OH**	0.40		−5.19	
